# Involvement of Yes-Associated Protein 1 Activation in the Matrix Degradation of Human-Induced-Pluripotent-Stem-Cell-Derived Chondrocytes Induced by T-2 Toxin and Deoxynivalenol Alone and in Combination

**DOI:** 10.3390/ijms25020878

**Published:** 2024-01-10

**Authors:** Li Liu, Huan Liu, Peilin Meng, Yanan Zhang, Feng’e Zhang, Yumeng Jia, Bolun Cheng, Mikko J. Lammi, Feng Zhang, Xiong Guo

**Affiliations:** 1 School of Public Health, Xi’an Jiaotong University Health Science Center, Key Laboratory of Trace Elements and Endemic Diseases, Collaborative Innovation Center of Endemic Disease and Health Promotion for Silk Road Region, Xi’an 710061, China; liuli0624@stu.xjtu.edu.cn (L.L.); huan.liu@xjtu.edu.cn (H.L.); mengpeilin@stu.xjtu.edu.cn (P.M.); sahalasanmao@126.com (Y.Z.); fenge0929@stu.xjtu.edu.cn (F.Z.); jia.yu.meng@163.com (Y.J.); boluncheng@xjtu.edu.cn (B.C.); mikko.lammi@umu.se (M.J.L.); 2 School of Nursing, Lanzhou University, Lanzhou 730000, China; 3 Department of Integrative Medical Biology, University of Umeå, 901 87 Umeå, Sweden

**Keywords:** Kashin–Beck disease, T-2 toxin, DON, hiPSCs, YAP

## Abstract

T-2 toxin and deoxynivalenol (DON) are two prevalent mycotoxins that cause cartilage damage in Kashin–Beck disease (KBD). Cartilage extracellular matrix (ECM) degradation in chondrocytes is a significant pathological feature of KBD. It has been shown that the Hippo pathway is involved in cartilage ECM degradation. This study aimed to examine the effect of YAP, a major regulator of the Hippo pathway, on the ECM degradation in the hiPS-derived chondrocytes (hiPS-Ch) model of KBD. The hiPS-Ch injury models were established via treatment with T-2 toxin/DON alone or in combination. We found that T-2 toxin and DON inhibited the proliferation of hiPS-Ch in a dose-dependent manner; significantly increased the levels of YAP, SOX9, and MMP13; and decreased the levels of COL2A1 and ACAN (all *p* values < 0.05). Immunofluorescence revealed that YAP was primarily located in the nuclei of hiPS-Ch, and its expression level increased with toxin concentrations. The inhibition of YAP resulted in the dysregulated expression of chondrogenic markers (all *p* values < 0.05). These findings suggest that T-2 toxin and DON may inhibit the proliferation of, and induce the ECM degradation, of hiPS-Ch mediated by YAP, providing further insight into the cellular and molecular mechanisms contributing to cartilage damage caused by toxins.

## 1. Introduction

Kashin–Beck disease (KBD) is a chronic, endemic bone and joint disease commonly occurring in preadolescents and adolescents in certain regions of China and other parts of Asia [1]. It is characterized by the degeneration of joint cartilage and bone, leading to pain, stiffness, and reduced mobility. While the exact cause of KBD is still insufficiently clear, previous studies have identified T-2 toxin and deoxynivalenol (DON) as potential risk factors. These are highly toxic mycotoxins produced by certain species of Fusarium fungi and lead to various toxicities, such as immunotoxicity, hepatotoxicity, and neurotoxicity [2]. Our group’s previous findings have confirmed that T-2/DON inhibits proliferation and induces apoptosis in C28/I2 cells, possibly through a reactive oxygen species (ROS)-mediated mitochondrial pathway [3]. However, the mechanisms underlying the effect of toxins on cartilage extracellular matrix (ECM) degradation have not yet been fully elucidated.

ECM is composed predominantly of collagen, non-collagenous glycoproteins, hyaluronan, and proteoglycans. Cartilage matrix-related proteins such as SOX9, COL2A1, MMP13, and aggrecan (ACAN) play a crucial role in the formation and maintenance of cartilage tissue. SOX9, a marker of immature chondrocytes, is an indispensable transcriptional factor that promotes chondrocyte differentiation by directly activating the chondrocyte-specific genes COL2A1 and ACAN during endochondral ossification. SOX9 is crucial for the maintenance of the ECM in articular cartilage, and the deletion of SOX9 results in impaired chondrocyte differentiation and a reduced production of important cartilage matrix components. This ultimately affects the overall structure and function of cartilage, leading to various cartilage-related disorders, such as skeletal dysplasia and osteoarthritis (OA). COL2A1 plays a crucial role in maintaining the structural integrity and function of cartilage tissue, and the cleavage of COL2A1 is considered a critical early event of OA. Collagenase 3 (MMP13) is the key enzyme in the cleavage of type II collagen and plays a significant role in cartilage collagen degradation in OA joints [4]. Understanding the intricate interplay between these genes and their role in chondrocyte function is crucial for developing therapeutic strategies to treat cartilage-related disorders, including OA and KBD. In the present study, the expression levels of MMP13, SOX9, and the ECM components COL2A1 and ACAN were tested as the ECM markers for induced pluripotent stem cell (iPSC)-derived chondrocytes (hiPS-Ch) in vivo.

Yes-associated protein 1 (YAP), a transcriptional coactivator, is a downstream gene of the Hippo signaling pathway. It is found both in the cytoplasm and in the nucleus, with the latter being the key functional form. Recent studies have indicated that the Hippo/YAP signaling pathway may play a significant role in the occurrence, development, and cartilage repair of cartilage diseases. The activation of YAP is crucial for maintaining cartilage integrity and attenuating cartilage degradation during OA progression. Conversely, the deletion of YAP in chondrocytes can promote cartilage disruption [5]. However, some studies have suggested contradictory conclusions regarding the role of YAP in cartilage development. Namely, some studies have shown that nuclear YAP negatively regulates chondrocyte differentiation [6]. Reduced YAP expression helps to maintain the chondrogenic phenotype while inhibiting chondrocyte proliferation [7]. The diverse role of YAP might be attributed to its different locations and interaction with other proteins. A previous study has suggested that YAP interacts with SOX6 and COL10A1 to regulate chondrocyte differentiation at multiple steps during endochondral ossification and bone repair [8]. According to Liu et al., the expression of YAP increases the expression and nuclear localization of β-catenin, and the transcriptional YAP/β-catenin complex could upregulate osteogenic, chondrogenic, and angiogenic factors [9]; this indicates the direct role of YAP in maintaining bone homeostasis and regulating chondrocyte function. Therefore, it is important to determine whether and how YAP regulates chondrogenesis, cartilage development, and homeostasis. However, whether YAP plays a role in the ECM degradation of hiPS-Ch treated with T-2 toxin/DON remains unclear. 

Despite great efforts, the exact causes and pathogenesis of KBD remain poorly understood, given the limited availability of suitable disease models. The most commonly used disease models are derived from animal species, such as monkeys, rats, pigs, and chickens [10]. However, the identified susceptible animal models of KBD have certain limitations; namely, they fail to fully replicate the same pathological changes in the cartilage of humans with KBD, especially the classical deep-zone chondronecrosis changes in the growth and development stage of the cartilage. In recent years, there has been an increasing trend of utilizing iPSCs to model human disorders. This approach has emerged as a valuable alternative to traditional animal models, which may have limitations in terms of accurately representing human physiology and disease processes. A notable advantage of iPSCs is their ability to be derived from individuals with specific diseases, enabling researchers to investigate the pathology and underlying mechanisms of these conditions.

In the present study, we examined the expression levels of chondrogenic markers in hiPS-Ch treated with T-2 toxin/DON alone or in combination. The objective of this study was to investigate the effects of T-2 toxin and DON on the proliferation and ECM composition of hiPS-Ch. Additionally, since YAP plays a crucial role in chondrocyte proliferation and differentiation, we aimed to investigate the involvement of YAP protein in chondrocyte injury caused by T-2 toxin and DON exposure. The results could provide valuable insights into the potentially toxic effects of these mycotoxins on chondrocytes and the integrity of the ECM.

## 2. Results

### 2.1. Identification of hiPSC Pluripotency and hiPS-Ch Chondrogenic Markers

**Figure 1:**
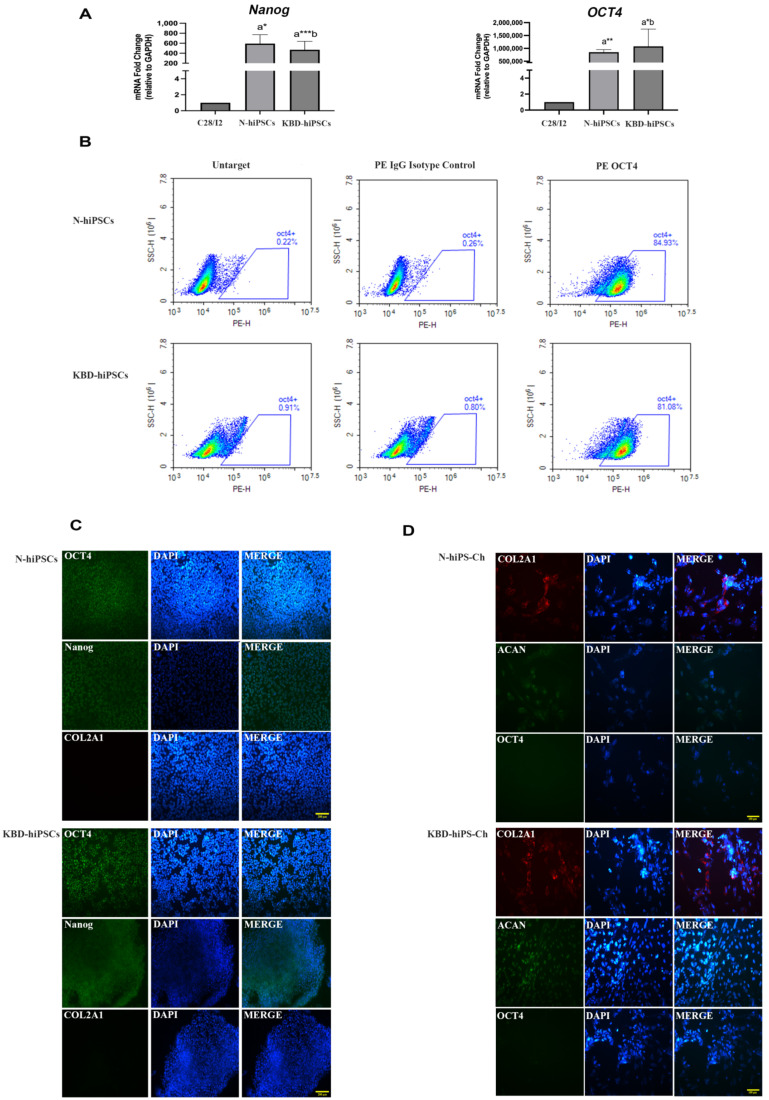
Pluripotency markers of hiPSCs and chondrogenic markers of hiPS-Ch. (**A**,**B**) The expression of the pluripotency markers OCT4 and Nanog in hiPSCs was determined via qRT-PCR and FCM analysis. (**C**) Immunocytochemistry identification of hiPSC colonies (Nanog, OCT4, and COL2A1), original magnification: 10×, scale bar = 200 µm. (**D**) Immunocytochemistry identification of hiPS-Ch (ACAN, COL2A1, and OCT4). Original magnification: 20×, scale bar = 100 µm. ^a^ Compared with C28/I2 human chondrocyte cell line, ^b^ compared with N-hiPSCs. * *p* < 0.05; ** *p* < 0.01; *** *p* < 0.001.

The RT-PCR results showed that the mRNA expressions of the pluripotency genes *OCT4* and *Nanog* were higher in hiPSCs generated from normal individuals and KBD patients (N-hiPSCs and KBD-hiPSCs, respectively) compared with the C28/I2 human chondrocyte cell line (all *p* < 0.001, Figure 1A). The expression of OCT4 in hiPSCs was also confirmed using flow cytometry (FCM) (Figure 1B), indicating that more than 80% of the KBD-hiPSCs and N-hiPSCs used in this study expressed OCT4 (Figure 1B, Supplementary File S1). Furthermore, IF staining revealed that KBD-iPSCs and N-iPSCs strongly expressed the intracellular pluripotency markers OCT4 and Nanog (Figure 1C), while chondrogenically differentiated KBD-hiPS-Ch and N-hiPS-Ch strongly expressed the chondrogenic markers COL2A1 and ACAN (Figure 1D). Collectively, these data demonstrate that the KBD-hiPSCs and N-hiPSCs used in this experiment were all pure hiPSC cell lines, with no other cell pollution. In addition, the hiPSCs were successfully differentiated into hiPS-Ch in the current study.

### 2.2. T-2 Toxin and DON Alone and in Combination Negatively Regulate hiPS-Ch Proliferation and Affect Chondrocyte Phenotype

**Figure 2:**
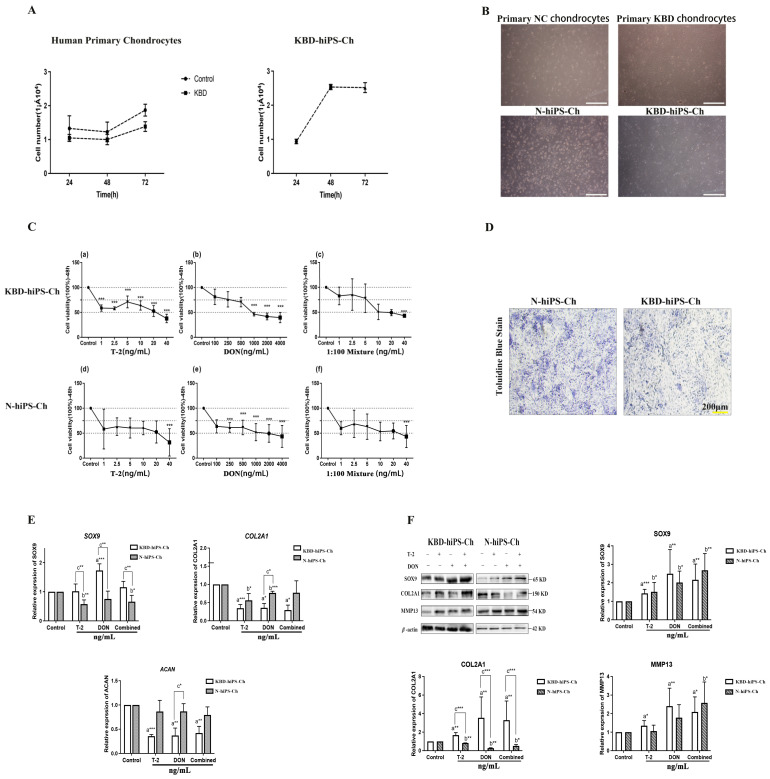
The effect of T-2 toxin/DON alone or in combination on hiPS-Ch proliferation and ECM synthesis. (**A**) The curve growth of the primary chondrocytes of KBD, control subjects, and KBD-hiPS-Ch. (**B**) The cell morphology of the primary chondrocytes of KBD, control subjects, KBD-hiPS-Ch, and N-hiPS-Ch. Original magnification: 4×, scale bar = 500 µm. (**C**) The Cell Counting Kit-8 assay examined the proliferation of hiPS-Ch induced by T-2 toxin, DON, and mixtures of T-2 toxin and DON for 48 h. a–c represent the T-2 toxin, DON, and 1:100 mixture of T-2 toxin and DON on KBD- hiPS-Ch proliferation; d–f represent the T-2 toxin, DON, and 1:100 mixture of T-2 toxin and DON on N-hiPS-Ch. (**D**) Toluidine blue staining was performed for N-hiPS-Ch and KBD-hiPS-Ch. Original magnification: 10×, scale bar = 200 µm. (**E**) The mRNA and protein (**F**) expression levels of chondrogenic markers of hiPS-Ch treated with T-2 toxin/DON. ^a^ Compared with an untreated group of KBD-hiPS-Ch, ^b^ compared with an untreated group of N-hiPS-Ch, ^c^ compared with a treated group of KBD-hiPS-Ch. * *p* < 0.05; ** *p* < 0.01; *** *p* < 0.001. N =  3 independent biological replicates per group. Data are presented as the mean  ±  SE. *p*-values were calculated via a one-way ANOVA test. In addition, the variance between KBD-hiPS-Ch and N-hiPS-Ch was detected.

The hiPS-Ch were further treated with T-2 toxin/DON alone or in combination, and the expression of key chondrogenic markers, including SOX9, COL2A1, ACAN, and MMP13, was tested. The growth curve (Figure 2A) and cell morphology (Figure 2B) of primary chondrocytes and hiPS-Ch show good growth in the chondrocytes. The CCK8 assay showed that T-2, DON, and their mixture all suppressed cell proliferation/cell viability in a dose-dependent manner in KBD-hiPS-Ch and N-hiPS-Ch (Figure 2C) (all *p* < 0.001). Based on the results of the CCK8 assay, 5 ng/mL T-2, 500 ng/mL DON, and the combined toxicity of T-2+DON (5 + 500) ng/mL was optimal for hiPS-Ch stimulation; thus, these amounts were selected for subsequent analysis. In addition, the results of toluidine blue staining showed that both hiPS-Ch models expressed chondrogenesis-associated ECM (Figure 2D).

We further examined the mRNA and protein expression levels of chondrocyte-specific marker genes in T-2/DON-induced KBD-hiPS-Ch and N-hiPS-Ch via qRT-PCR (Figure 2E) and Western blotting analysis (Figure 2F). Interestingly, the mRNA expression of *SOX9* was decreased in N-hiPS-Ch treated with T-2 alone and T-2 toxin/DON combination, while DON alone increased the expression of *SOX9* in KBD-hiPS-Ch. RT-PCR demonstrated that T-2 and DON decreased the mRNA expression levels of *COL2A1* and *ACAN* (Figure 2E) in both hiPS-Ch models. These results suggest that treatment with T-2 toxin and DON, either alone or in combination, has a different effect on chondrogenic maintenance in N-hiPS-Ch and KBD-hiPS-Ch.

In the present study, the protein expression levels of the differentiation markers SOX9 and MMP13 were increased in both hiPS-Ch groups (Figure 2F, Supplementary File S2). The toxin-induced increase in SOX9 in hiPS-Ch suggests that chondrocytes are constantly struggling with their degradation process. However, in the T-2/DON-treated groups, the protein levels of COL2A1 were decreased in N-hiPS-Ch and increased in KBD-hiPS-Ch compared with the non-treated group (Figure 2F, Supplementary File S2). The decline in COL2A1 indicates that cartilage degradation is irreversible. We can conclude that these two toxins significantly reduced the proliferation of hiPS-Ch and downregulated the expression of the chondrogenic markers COL2A1 and ACAN, indicating that T-2/DON negatively affects chondrocyte proliferation and ECM synthesis in vivo.

### 2.3. T-2 Toxin and DON Alone and in Combination Active the Expression of YAP in hiPS-Ch 

**Figure 3:**
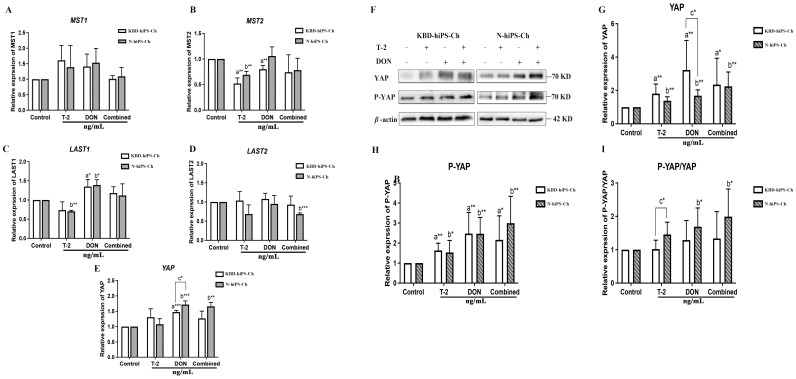
The effect of T-2 toxin/DON alone or in combination on YAP expression levels in hiPS-Ch. (**A**–**E**) Detection of Hippo/YAP pathway mRNA expression in hiPS-Ch treated with T-2 toxin/DON via quantitative qRT-PCR. (**F**–**I**) Detection of YAP, pYAP in hiPS-Ch treated with T-2 toxin/DON via Western blotting. ^a^ Compared with an untreated group of KBD-hiPS-Ch, ^b^ compared with an untreated group of N-hiPS-Ch, ^c^ compared with a treated group of KBD-hiPS-Ch. * *p* < 0.05; ** *p* < 0.01; *** *p* < 0.001. N =  3 independent biological replicates per group. Data are presented as the mean  ±  SE. *p*-values were calculated via one-way ANOVA test. In addition, the variance between KBD-hiPS-Ch and N-hiPS-Ch was detected.

We showed above that T-2 toxin/DON can negatively affect the chondrogenic phenotype by inhibiting the gene expression of COL2A1 and ACAN. We further tested the mRNA and protein expression levels of the YAP/Hippo pathway in T-2/DON-induced KBD-hiPS-Ch and N-hiPS-Ch. Quantitative RT-PCR showed that the *YAP* mRNA expression level was significantly higher in the DON-treated groups compared with the untreated groups in both hiPS-Ch models. In addition, there was a significant difference in *YAP* mRNA expression between KBD-hiPS-Ch and N-hiPS-Ch (*p* < 0.05) (Figure 3E). A high mRNA expression level of *YAP* was observed in the DON and combined groups in both hiPS-Ch models (Figure 3E). Western blotting showed that the protein expression levels of total YAP (Figure 3G, Supplementary File S2) and P-YAP (ser 127) (Figure 3H, Supplementary File S2) were significantly higher in the toxin-treated groups than in the untreated group (*p* < 0.05). In addition, the P-YAP/YAP protein expression level was higher in the toxin-treated group in the N-hiPS-Ch group (*p* < 0.05) (Figure 3I, Supplementary File S2). Our results demonstrate that T-2 and DON could upregulate the expression levels of total YAP and inhibit the YAP/Hippo pathway in hiPS-Ch.

### 2.4. T-2 Toxin and DON Alone and in Combination Affect the Chondrogenic Phenotype of hiPS-Ch via the Activation of YAP

**Figure 4:**
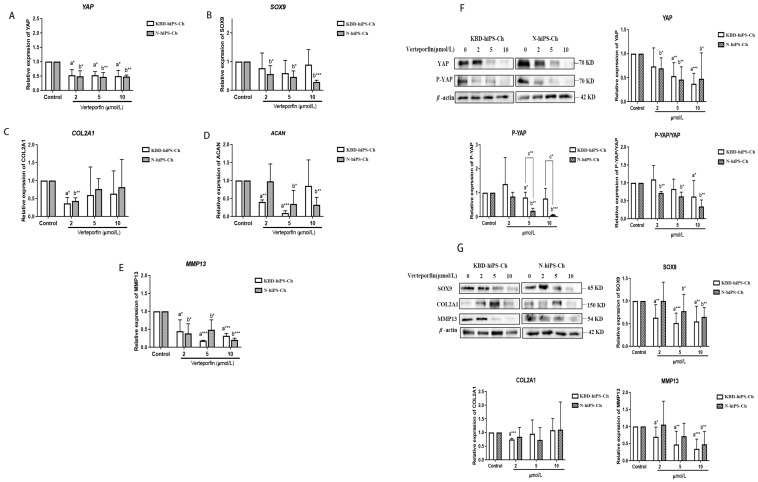
Effects of different concentrations of YAP inhibitors on chondrocyte phenotypic-related protein expressions and Hippo/YAP pathway expressions. (**A**–**E**) Detection of chondrocyte phenotypic-related protein expressions and Hippo/YAP pathway expressions in hiPS-Ch treated with different concentrations of verteporfin via quantitative RT-PCR. (**F**,**G**) Detection of YAP, pYAP, and chondrocyte phenotypic-related protein in hiPS-Ch treated with different concentrations of verteporfin via Western blotting. ^a^ Compared with an untreated group of KBD-hiPS-Ch, ^b^ compared with an untreated group of N-hiPS-Ch, ^c^ compared with a treated group of KBD-hiPS-Ch. * *p* < 0.05; ** *p* < 0.01; *** *p* < 0.001. N =  3 independent biological replicates per group. Data are presented as the mean  ±  SE. *p*-values were calculated via a one-way ANOVA test. In addition, the variance between KBD-hiPS-Ch and N-hiPS-Ch was detected.

We showed above that T-2 toxin/DON upregulated the expression levels of YAP and SOX9. It has been found that YAP-SOX9 feedback plays an important role in disease mechanisms [11]. Verteporfin has recently been identified as an inhibitor of the interaction of YAP with TEAD. Therefore, to comprehensively evaluate the association between the YAP and SOX9 in hiPS-Ch, we further used different concentrations of verteporfin to inhibit the expression of YAP and test the expression levels of the chondrogenic markers. As shown in Figure 4, decreased mRNA (Figure 4A–E) and protein (Figure 4F,G, Supplementary File S2) levels of YAP were detected with the increased verteporfin concentration (all *p* < 0.05). In addition, verteporfin significantly downregulated the expression level of SOX9 in KBD-hiPS-Ch and N-hiPS-Ch (Figure 4B,G, Supplementary File S2) (all *p* < 0.05). The downregulation of YAP resulted in significantly reduced expression levels of MMP13 and SOX9 (Figure 4G, Supplementary File S2). Taken together, these data suggest that YAP may affect the chondrogenic phenotype of hiPS-Ch.

### 2.5. Nuclear Translocation of YAP in hiPS-Ch Induced by T-2 Toxin/DON Alone or in Combination

**Figure 5:**
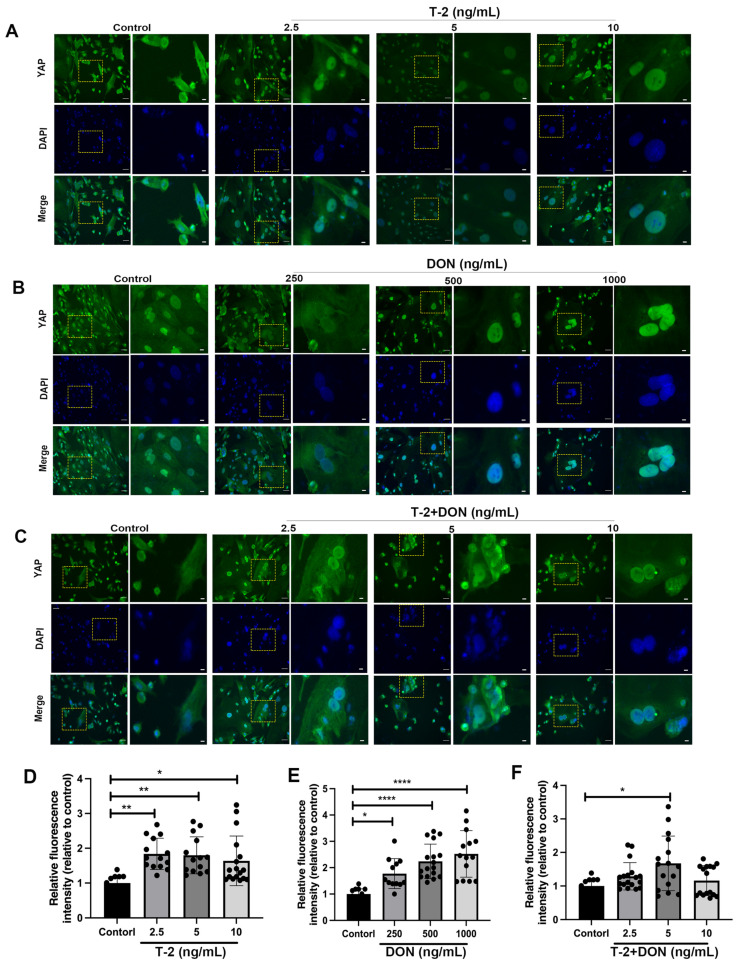
YAP localization under the effect of different concentrations of T-2 toxin/DON alone or in combination. (**A**–**C**) Altered nuclear displacement of YAP induced by different concentrations of T-2 toxin/DON alone or in combination. (**D**–**F**) Relative fluorescence expression of YAP induced by different concentrations of T-2 toxin/DON alone or in combination. Scale bars = 50 μm for original images and 10 μm for boxed images. N =  3 independent biological replicates per group. Data are presented as the mean  ±  SE. *p*-values were calculated via a one-way ANOVA test, compared with a non-treated group (**D**–**F**). * *p* < 0.05; ** *p* < 0.01; **** *p* < 0.0001.

We further performed immunofluorescent staining to determine the location of YAP expression. The green fluorescence depicts the positive expression of the YAP protein. As revealed via immunofluorescent staining, the YAP protein was expressed both in the nucleus and in the cytoplasm. The fluorescence in the nucleus was gradually increased and the green fluorescence in the cytoplasm was gradually decreased with the increasing T-2 toxin and DON concentrations. The expression of YAP increased with the increased concentrations of T-2 toxin (*p* < 0.05) (Figure 5A,D) (*p* < 0.01) and DON (Figure 5B,E) (*p* < 0.001). At the highest concentrations of the toxin group, we found that the YAP protein was predominantly localized in the nucleus of hiPS-Ch and was almost absent in the cytoplasm. However, as shown in Figure 5C, the combined toxicity of T-2 and DON did not significantly change YAP expression (*p* < 0.05). Our observations confirmed an enhanced expression of YAP in N-hiPS-Ch induced by T-2 toxin, DON, and combined toxins.

## 3. Discussion

KBD is an endemic disease characterized by necrosis and apoptosis in the deep zone of the growth and articular cartilage. In this study, we used the KBD and normal hiPS-Ch constructed by our group to represent the KBD/normal disease model, which has a genetic background for KBD [12] and is superior to animal models. We tested the effect of T-2 toxin and DON on the proliferation and ECM component expression of hiPS-Ch and evaluated the possible functional role of YAP in toxin-induced cartilage injury. Our results support that T-2 toxin/DON inhibits chondrocyte proliferation and negatively regulates the chondrocyte phenotype, leading to ECM degradation in hiPS-Ch, which is possibly mediated in part by YAP.

T-2 toxin and DON are the most important food contaminants, given their frequent occurrence and high concentrations in cereals. T-2 toxin, produced by various Fusarium species, is considered the most toxic fungal secondary metabolite among trichothecenes. DON is the most commonly found mycotoxin contaminant in food and feed. Exposure to DON can lead to a range of toxic effects in both humans and animals. T-2 toxin and DON inhibit the synthesis of proteins and promote the generation of ROS [13], affecting various cell types and organs [14,15,16]. Although both T-2 toxin and DON are considered toxic substances, T-2 toxin has a higher toxicity than DON. Additionally, T-2 toxin and DON exhibit distinct effects in certain aspects, such as stress-granule formation [17]. The potential adverse effects of the interactions between T-2 toxin and DON when present simultaneously in a mixture have attracted widespread attention [18]. It has been shown that T-2 toxin and DON can cause chondrocyte injury by inhibiting protein synthesis, reducing the secretion of aggrecan, and accelerating its degradation [19]. A previous study suggested that miR-140 might be involved in T-2 toxin-induced degradation of the ECM of articular cartilage [20]. Animal models have suggested that T-2 toxin may cause KBD in low-Se conditions [21]. However, the toxic effects of T-2 toxin and DON on the ECM synthesis of hiPS-Ch remain largely unknown. The present study aimed to analyze the toxicity of T-2 toxin and DON, alone and in combination, on cartilage degradation. We showed that T-2 toxin and DON inhibited the proliferation of hiPS-Ch in a dose-dependent manner and decreased the mRNA and protein expression of the ECM synthetic markers COL2A1 and ACAN, indicating that T-2 toxin and DON induce the ECM degradation of hiPS-Ch. However, SOX9 expression was increased with toxic treatment, suggesting that the cells might be involved in cartilage damage repair. Taken together, our results show that the T-2 toxin/DON alone or in combination inhibited hiPS-Ch proliferation, decreasing the matrix-associated proteins and leading to chondrocyte injury.

YAP is a transcriptional coactivator. It plays an important role in organ size control and can regulate the proliferation, survival, apoptosis, and differentiation of cells, and tissue homeostasis [22]. Moreover, YAP is an effector of the Hippo signaling pathway, localized to both the cytoplasm and the nucleus. The sequestration of phosphorylated YAP in the cytoplasm inhibits its transcriptional activity. In contrast, the inactivation of the Hippo pathway increases YAP/TAZ nuclear translocation. Subsequent studies have demonstrated that YAP interacts with many transcription factors for the crosstalk with different signaling pathways and serves as a regulator to integrate multiple signaling cascades during many biological processes. YAP plays an essential role in the proliferation and differentiation of chondrocytes, as well as in phenotype maintenance. Changes in YAP localization in chondrocytes likely correlate with changes in the chondrogenic phenotype. A large body of experimental evidence suggests that YAP is overexpressed in OA animal model tissues [23,24] and human OA tissues [23]. A previous study showed that YAP negatively regulates ATDC5 cell chondrogenic and hypertrophic differentiation, partly by activating the β-catenin signaling pathway [25]. A recent study has also shown that YAP expression is upregulated after injury in the Gdf5-lineage cells in the synovium, as well as during cartilage repair [26]. Yu et al. reported that the expression level of YAP protein in the nucleus increased in ATDC5 cells after treatment with fluoride, as did the expression of MMP13, indicating the association between the Hippo pathway and the ECM degradation of chondrocytes [27]. Nevertheless, the role of YAP in the maintenance of the chondrocyte phenotype is still controversial. Deng et al. found that the activation of YAP in chondrocytes attenuated OA progression. On the other hand, the loss of YAP exaggerated cartilage destruction during OA, indicating that YAP is necessary and sufficient to protect against cartilage degradation in OA conditions [5]. Li et al. demonstrated the protective role of YAP in T-2 toxin-induced cartilage damage [28]. In the present study, YAP mRNA and protein levels increased when hiPSCs were differentiated into hiPS-Ch based on the standard protocol and treated with T-2 toxin and DON, alone or in combination. Moreover, the expression levels of subsequent chondrogenic markers COL2A1 and ACAN decreased, indicating that YAP is involved in the ECM degradation of hiPS-Ch induced by toxins. To comprehensively uncover the effect of T-2 toxin/DON on the effect of YAP nuclear/cytoplasmic expression, we further treated the hiPS-Ch with different concentrations of toxins. We showed that T-2 toxin and DON activated the expression of the YAP protein and increased the YAP nuclear translocation. Overactivated YAP leads to the dedifferentiation of chondrocytes, destroys the normal phenotype and function of chondrocytes, and leads to degeneration and injury in cartilage tissue.

Studies have revealed that SOX9 is a master chondrogenic transcription factor and is essential for chondrocyte differentiation and cartilage formation [29]. The expression of SOX9 starts in chondroprogenitor cells and is maintained in proliferating chondrocytes [30]. It cooperatively interacts with transcriptional factors Sox5 and Sox6 to promote both chondrocyte proliferation and differentiation [31]. YAP can regulate the transcription of SOX9, and YAP-driven SOX9 expression is involved in cancer development [32,33]. However, the coordination between YAP and SOX9 in chondrocytes, and the mechanism by which they regulate chondrocytes together, still need to be defined. To clarify this question, we further treated the hiPS-Ch with different concentrations of the small-molecule inhibitor of YAP, verteporfin. Verteporfin significantly decreased the expression levels of YAP and SOX9. Previous studies have shown that the role of the YAP1/TAZ-TEAD complex as a transcriptional activator or repressor of SOX9 expression depends on the tissue-specific context [11]. Given the important role of YAP and SOX9 in chondrocyte proliferation and the maintenance of the chondrocyte phenotype, our preliminary results indicate that the expression levels of YAP and SOX9 are significantly correlated with each other, suggesting that YAP might regulate the expression of SOX9 in hiPS-Ch. However, further molecular experiments are needed for validation.

Chondroitin sulfate is a polysaccharide found in cartilage and a major component of cartilage ECM. Chondroitin sulfate is demonstrated to be effective in alleviating symptoms and improving the dysfunction of KBD [34] and OA [35]. And the biotechnological production of chondroitin sulfate and its oligosaccharides is a promising method for addressing the drawbacks associated with animal-derived chondroitin sulfate [36] and could be used for novel biomaterials for cartilage regeneration [37]. Previous studies showed that the presence of chondroitin sulfate and, mostly, unsulfated biotechnological chondroitin improved the mechanical features of hydrogels and prompted the differentiation of MSCs through the over-expression of chondrogenic differentiation genes [38], making the chondroitin sulfate-based biomimetic scaffold a promising strategy in the field of cartilage and bone repair. The next stage of our study was to identify a new biomaterial to be a scaffold for the hiPS-Ch 3D culture and use it for cartilage regeneration.

Several main limitations should be noted. First, due to the limited sample size, KBD-hiPSCs and N-hiPSCs were obtained from only one KBD patient and one normal patient. Therefore, larger sample sizes are needed to further validate the results. Second, the effect of toxins on the chondrogenesis of hiPS-Ch was studied in a two-dimensional culture system. To accurately locate the site of toxin-induced cartilage damage and elucidate the histological features of the affected area, future studies using a three-dimensional pellet culture system are warranted. Third, FCM should be applied to confirm the expression of all pluripotency markers in further analysis.

## 4. Materials and Methods

### 4.1. Chemicals and Culturing of C28/I2 Cells

T-2 toxin (No. MSS023) and DON (No. MSS011) were purchased from Pribolab (Qingdao, China). The human chondrocyte cell line (C28/I2), as described in the previous study [39], was provided by Prof. Mary B. Goldring. Culturing of the cells was performed in DMEM/F12 containing 10% fetal bovine serum (Hyclone, Logan, UT, USA), penicillin (100 U/mL), and streptomycin (100 mg/L) at 37 °C in 5% CO_2_.

### 4.2. Primary Cartilage Tissue Collection and Chondrocyte Isolation

A total of 3 samples of human articular cartilage from individuals with KBD and 3 samples from normal controls were included in the present study. Both KBD and normal subjects were of Chinese Han lineage and were matched for age. The patients with KBD were diagnosed in strict accordance with the national diagnostic criteria of KBD in China [WS/T 207-2010]. The articular cartilage samples from KBD were collected from individuals who had undergone knee arthroplasty, while healthy control specimens were obtained from patients who had suffered trauma or amputation due to an accident. Subjects were excluded from this study if they had a current or previous history of osteoarticular diseases (e.g., rheumatoid arthritis, gout, skeletal fluorosis), macrosomia, osteochondrodysplasia, chronic diseases (e.g., hypertension, diabetes, coronary heart disease), or if they had received any treatment within the past 6 months. Clinical information was collected from patient records. All donors signed a written informed consent form.

Chondrocytes were isolated as follows: articular cartilage specimens were washed twice with sterile phosphate-buffered saline (PBS) with antibiotics (penicillin and streptomycin) and subjected to enzymatic digestion with 0.25% trypsin solution for up to 30 min, and the specimens were cut into pieces (1–3 mm^3^). We rinsed the minced cartilage once with PBS and transferred the pieces of cartilage to a 50 mL centrifuge tube, and the cells were digested in basal media supplemented with 0.2% type II collagenase at 37 °C overnight. The isolated chondrocytes were filtered through 70 mM nylon filters, and the cell suspensions were centrifuged at 1000× *g* for 5 min. After discarding the supernatant, the cells were resuspended into 5 mL of complete culture medium and further cultured in a CO_2_ incubator at 37 °C. The medium was changed every other day.

### 4.3. Culturing and Differentiation of hiPSCs

As shown in the previous literature published by our group [12], we have successfully generated the first hiPS-Ch from dermal fibroblasts of KBD patients and normal patients, which can provide the disease models and chondrocyte sources for in-depth mechanism studies of early KBD in vitro. The detailed reprogramming methods to generate KBD-hiPSCs and N-iPSCs have been described previously [12]. Both hiPSCs were differentiated into chondrocytes through a 2-week culture based on improved direct differentiation methods from the existing studies [40,41]. Cells were seeded in a 6-well plate and incubated at 37 °C in a humidified incubator containing 5% CO_2_. Briefly, for growth medium, hiPSCs were seeded on plates coated with Matrigel™ (Corning) and maintained in mTeSR-1 medium (StemCell Technologies, Vancouver, BC, Canada). Subsequently, the hiPSCs were subjected to chondrogenic differentiation in E6 medium (DMEM/F12 (HyClone, Logan, UT, USA), containing 1% insulin, 1% transferrin, 1% sodium selenite, and 1% vitamin C) with 25 ng/mL Wnt3a (PeproTech, Cranbury, NJ, USA) and 50 ng/mL Activin A (PeproTech) for 3 days. It is worth noting that 20 ng/mL of FGF2 (PeproTech) was added to the medium on day 2, and 40 ng/mL of BMP4 (PeproTech) was added to the medium on day 3. Then, the medium was changed to E6 medium containing 20 ng/mL of FGF2, 40 ng/mL of BMP4, and 100 ng/mL of follistatin (PeproTech) on days 4–7. On day 8, follistain was removed from the medium. Finally, on days 9–14, the hiPSCs were maintained in an E6 medium with 10% fetal bovine serum (FBS) and 40 ng/mL GDF5 (PeproTech). The medium was changed every day during the differentiation period. The hiPS-Ch models were treated with several concentrations of individual and combined T-2 (1–20 ng/mL) and DON (100–2000 ng/mL) toxins, as well as 0.1–10 μM verteporfin (SML0534, Sigma-Aldrich, St. Louis, MO, USA) on day 14. At 48 h, after treatment, the cells were collected and subjected to subsequent analysis.

### 4.4. Flow Cytometry Assay

The intracellular pluripotent marker OCT4 for iPSCs was characterized via flow cytometry (FCM) using True-NuclearTM Transcription Factor Buffer Set (424401, Biolegend, San Diego, CA, USA). Cells were detached and digested into a single-cell suspension using trypsin. The cells were centrifuged and precipitated in a 15 mL tube. Then, they were fixed with True-NuclearTM 1× for 45 min at room temperature in the dark. We next added 2 mL buffer of True-NuclearTM 1×Perm to the tube, centrifuged the tubes, and discarded the supernatant. Next, we added 100 μL True-NuclearTM 1×Perm buffer and 5 μL PE anti-OCT4 (OCT3) antibody (653703, Biolegend) or the 0.625 μL PE Mouse IgG 2b isotype control (400313, Biolegend) to the samples, which were then incubated at room temperature for 30 min. Cell labeling was analyzed in a staining solution using NovoCyte Quanteon (https://www.agilent.com/en/product/research-flow-cytometry/flow-cytometers/flow-cytometer-systems/novocyte-quanteon-flow-cytometer-systems-4-lasers-984692, 3 January 2024, Agilent, Santa Clara, CA, USA), and data were analyzed using NovoExpress software (Agilent, America, version 1.6.2).

### 4.5. MTT Cytotoxicity Assay

KBD-hiPS-Ch and N-hiPS-Ch were seeded in 96-well plates at a density of 1 × 10^4^ cells per well and treated with various concentrations of T-2 toxin (1, 2.5, 5, 10 ng/mL), DON (100, 250, 500, 1000 ng/mL), and 1:100 T-2+DON toxin (1 + 2.5, 2.5 + 250, 5 + 500, 10 + 1000 ng/mL) for 48 h. We used 0.1–10 μM verteporfin (Sigma, Cat.SML0534) to inhibit YAP protein expression in hiPS-Ch for 48 h. Cell viability was then tested using a cell-counting kit (CCK8, NCM Biotech, Suzhou, China). The absorbance at 450 nm was used to calculate relative viability using a microplate reader (Model 3550, Bio-Rad, Hercules, CA, USA).

### 4.6. Toluidine Blue Staining

KBD-hiPS-Ch and N-hiPS-Ch were seeded in 24-well plates. The cells were fixed with 4% paraformaldehyde (PFA) for 15 min at room temperature and washed with PBS twice. Then, the cells were stained with toluidine blue (TB) for 5 min and washed twice with distilled water. The staining results were observed under a fluorescence microscope (Leica Microsystems, Wetzlar, Germany) and photographed.

### 4.7. Immunofluorescence Staining

The localization of YAP expression in N-hiPS-Ch was determined via immunofluorescence staining. Briefly, cells were harvested on day 14 and were seeded in 24-well plates at 1 × 104 cells/cm^2^. After 48 h of treatment with low, medium, and high concentrations of T-2 toxin and DON alone or in combination, the cells were fixed with 4% paraformaldehyde for 15 min at room temperature and washed with PBS thrice. The samples were then treated with 0.1% Triton X-100/PBS for 30 min at room temperature and blocked with 1% bovine serum albumin (BSA)/0.3 M glycine at 4 °C for 60 min. Subsequently, the samples were incubated with rabbit anti-YAP antibody (13584-1-AP, 1:100, Proteintech) at 4 °C overnight, followed by secondary fluorescent conjugated secondary antibodies. Cell nuclei were counterstained with DAPI (C1005, Beyotime, Shanghai, China). In addition, the identification of hiPSCs and hiPS-Ch was performed using immunofluorescence staining. The samples were incubated with an anti-Nanog antibody (14295-1-AP, 1:100), anti-OCT4 antibody (ab181557, 1:250), anti-COL2A1 antibody (ab185430, 1:100), and anti-ACAN antibody (13880-1-AP, 1:100). Staining was observed using a fluorescence microscope (Leica Microsystems, Germany) and photographed.

### 4.8. RNA Isolation and qRT-PCR

Total RNA was extracted from monolayer cell cultures using TRIzol reagent (Invitrogen, Waltham, MA, USA) following the manufacturer’s instructions. Reverse transcription was performed with 500 ng of total RNA using PrimeScript™ RT Master Mix (Takara Bio, Kusatsu, Japan). qPCR was carried out using SYBR Premix Ex TaqTM (Takara, Japan) in triplicate on QuantStudio™ 6 Flex Real-Time PCR System (Applied Biosystems, Waltham, MA, USA). The thermal cycling conditions were set as follows: 30 s at 95 °C, followed by 40 cycles at 95 °C for 5 s, and 60 °C for 30 s. The primer sequences are shown in Table 1. Samples were normalized to the endogenous control glyceraldehyde 3-phosphate dehydrogenase (GAPDH). The fold difference was determined via the 2^−ΔΔCT^ method.

### 4.9. Western Blot

The cells were washed with PBS three times. The total proteins were isolated from the chondrocytes and isolated using the RIPA buffer supplemented with 1 mM of phenylmethanesulfonyl fluoride. The mixture was sonicated on ice for 10 min, and then centrifugation was conducted at 12,000 rpm at 4 °C for 15 min. The BCA protein assay kit (Solarbio, Beijing, China) was used to determine the protein concentration. Then, equivalent amounts of proteins were separately subjected to 10% sodium dodecyl sulfate–polyacrylamide gel electrophoresis (SDS-PAGE) and electrotransferred onto PVDF membranes (Millipore, USA). The membranes were blocked with sealing fluid (Shanghai Epizyme Biotech, Shanghai, China) for 1.5 h at room temperature and incubated with anti-SOX9 (YT4371, 1:500, Immunoway, Plano, TX, USA), anti-YAP (13584-1-AP,1:5000, Proteintech, Rosemont, IL 60018, USA), anti-Phospho-YAP (Ser127) (bsm-52214R, 1:500, Bioss, Woburn, MA, USA), anti-COL2A1 (M2139) (sc-52658, 1:500, Santa Cruz, Dallas, TX, USA), anti-MMP13 (ab51072, 1:500, Abcam, Cambridge, UK), and anti-*β*-actin (GB5003, 1:2000, Servicebio, Wuhan, China) at 4 °C overnight, followed by incubation with the corresponding secondary antibodies for one hour at room temperature. The membranes were then washed using tris-buffered saline with Tween-20 (TBST), and chemiluminescent signals were developed using an ECL kit (Millipore, Darmstadt, Germany) and were exposed to X-ray film. Thereafter, the gray value of the band, normalized to *β*-actin, was quantified using Image J (Version 1.52a).

### 4.10. Statistical Analysis

All experiments in the present study were performed in triplicate. All results were presented as mean ± standard deviation. Statistical analyses were performed in SPSS 26.0 and selected based on the data distribution. Statistical differences were assessed via Student’s *t*-test or one-way ANOVA or the rank-sum test. Significance was considered at a *p* value < 0.05.

## 5. Conclusions

In summary, T-2 toxin and DON inhibit the proliferation of, and induce ECM degradation in, hiPS-Ch, which is mediated by YAP. These findings expand our knowledge of the cellular and molecular mechanisms involved in the cartilage damage caused by T-2 toxin and DON.

## Figures and Tables

**Table 1:** List of primers used for PCR (5′-3′).

Gene	Forward Primer	Reverse Primer
ACAN	ACGAAGACGGCTTCCACCAG	TCGGATGCCATACGTCCTCA
COL2A1	AGACTGGCGAGACTTGCGTCTA	ATCTGGACGTTGGCAGTGTTG
SOX9	GGAGATGAAATCTGTTCTGGGAATG	TTGAAGGTTAACTGCTGGTGTTCTG
Nanog	CCTGTGATTTGTGGGCCTGA	CTCTGCAGAAGTGGGTTGTTTG
OCT4	GTGCCGTGAAGCTGGAGAA	TGGTCGTTTGGCTGAATACCTT
MMP13	TCCCAGGAATTGGTGATAAAGTAGA	GCATGACGCGAACAATACGG
MST1	GTGCTACACGATGGACCCAA	CACCCTCTTGCCACACTTCT
MST2	CACGATGTTGGAATCCGACTTG	GTCTTTGTACTTGTGGTGAGGTTG
LAST1	CTCCACCACCTCTCAACACTT	CTGCCAACAGGAACAGAACTAATG
LAST2	CTCCGCAAAGGGTACACTCA	TCTGTGGGAGTAGGTGCCAA
YAP	GTGGATGAGATGGATACAGGTGAT	CAGGAATGGCTTCAAGGTAGTCT
GAPDH	GCACCGTCAAGGCTGAGAAC	TGGTGAAGACGCCAGTGGA

## Data Availability

All data generated or analyzed during this study are included in this published article.

## Supplementary Materials

The supporting information can be downloaded at: https://www.mdpi.com/article/10.3390/ijms25020878/s1.

## Abbreviations

DON	deoxynivalenol
hiPSCs	human-induced pluripotent stem cells
hiPS-Ch	human-induced pluripotent stem chondrocyte
KBD	Kashin–Beck disease
OA	osteoarthritis
YAP	yes-associated protein 1
ACAN	aggrecan
FCM	flow cytometry
PFA	paraformaldehyde
MSC	mesenchymal stem cell
