# Supplementary material for: Involvement of Yes-Associated Protein 1 Activation in the Matrix Degradation of Human-Induced-Pluripotent-Stem-Cell-Derived Chondrocytes Induced by T-2 Toxin and Deoxynivalenol Alone and in Combination
Source: Int J Mol Sci. 2024 Jan 10;25(2):878. doi: 10.3390/ijms25020878 (PMC10815455; doi:10.3390/ijms25020878)
Supplement: Supplementary file 1 [file ijms-25-00878-s001.zip › Supplementary file S1.pdf]

Supplementary files for flow cytometry results of Figure 1.

(1) Normal-hiPSC-ch

1. Untarget

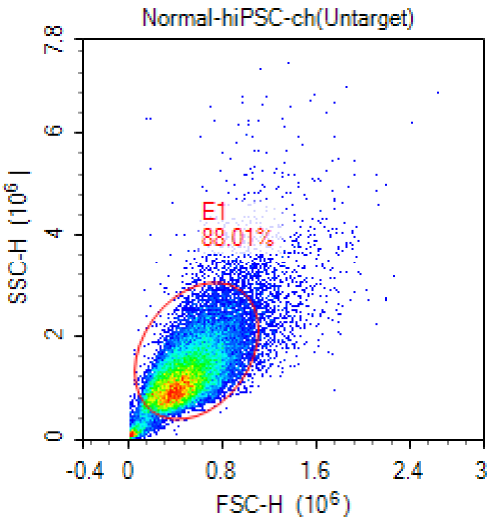

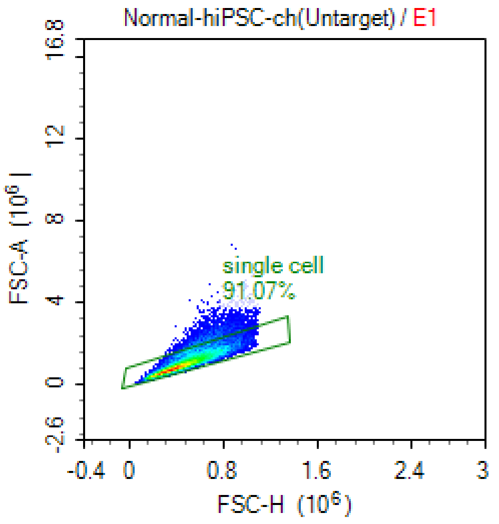

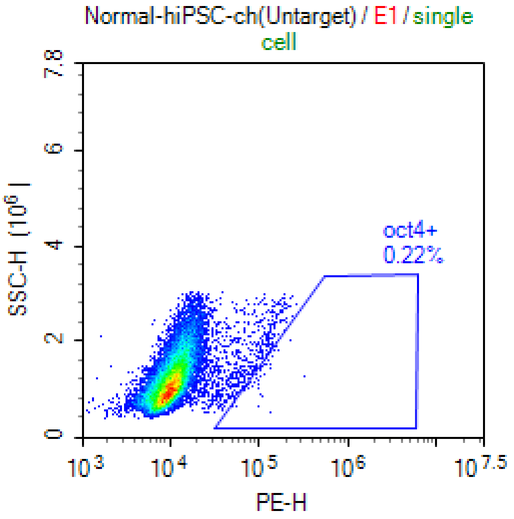

2. Isotype control

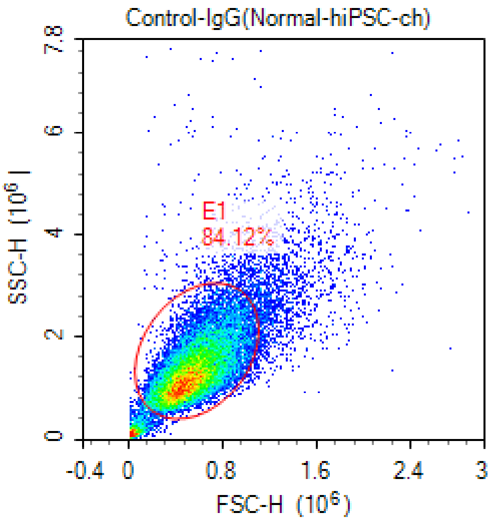
Control-IgG(Normal-hiPSC-ch)
E1
84.12%
SSC-H (10^6)
FSC-H (10^6)
7.8
6
4
2
0
-0.4
0
0.8
1.6
2.4
3

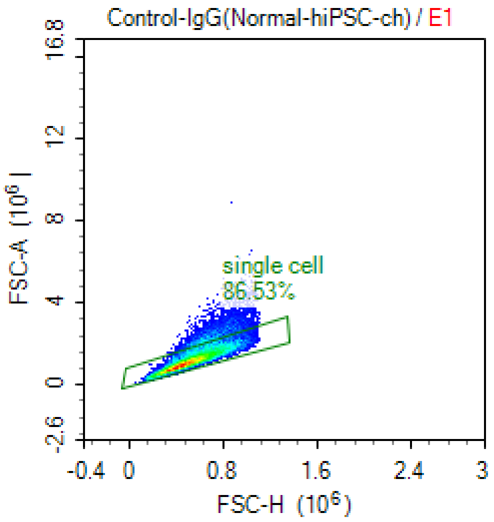
Control-IgG(Normal-hiPSC-ch) / E1
single cell
86.53%
FSC-A (10^6)
FSC-H (10^6)
16.8
12
8
4
0
-2.6
-0.4
0
0.8
1.6
2.4
3

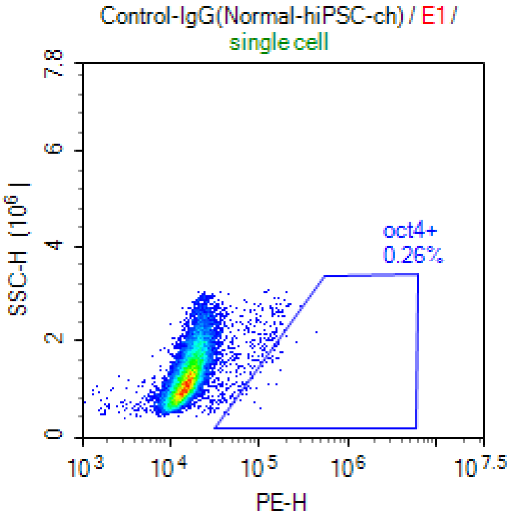
Control-IgG(Normal-hiPSC-ch) / E1 /
single cell
oct4+
0.26%
SSC-H (10^6)
PE-H
7.8
6
4
2
0
10^3
10^4
10^5
10^6
10^7.5

3. OCT4+

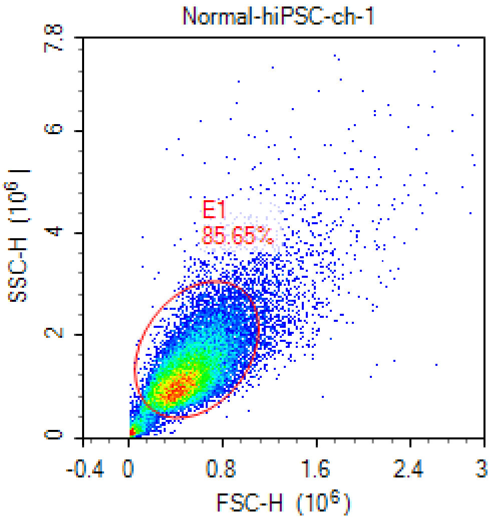
Normal-hiPSC-ch-1
E1
85.65%
SSC-H (10^6)
FSC-H (10^6)
7.8
6
4
2
0
-0.4
0
0.8
1.6
2.4
3

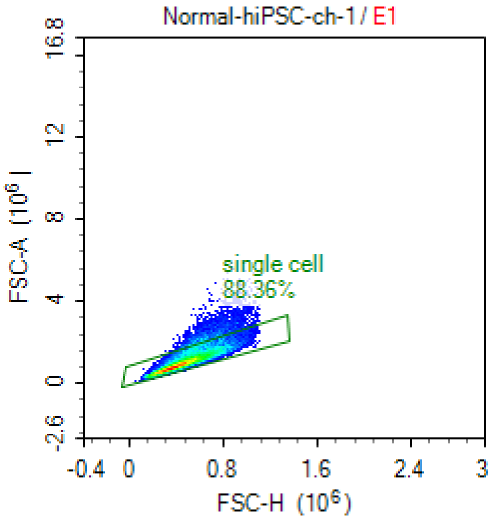
Normal-hiPSC-ch-1 / E1
single cell
88.36%
FSC-A (10^6)
FSC-H (10^6)
16.8
12
8
4
0
-2.6
-0.4
0
0.8
1.6
2.4
3

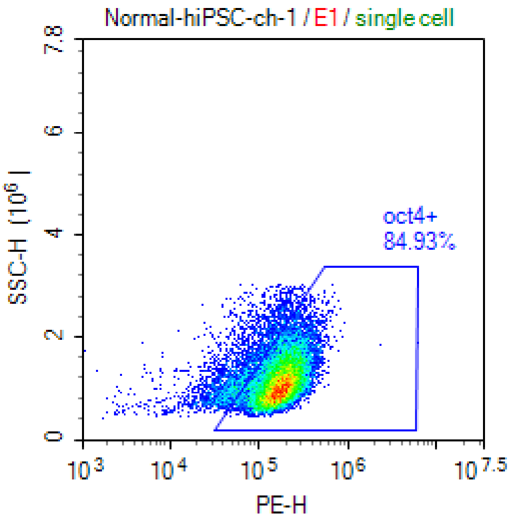

(2) KBD-hiPSCs-ch

1. Untarget

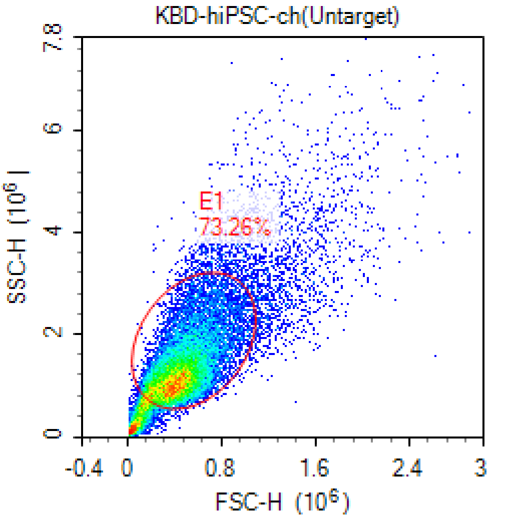

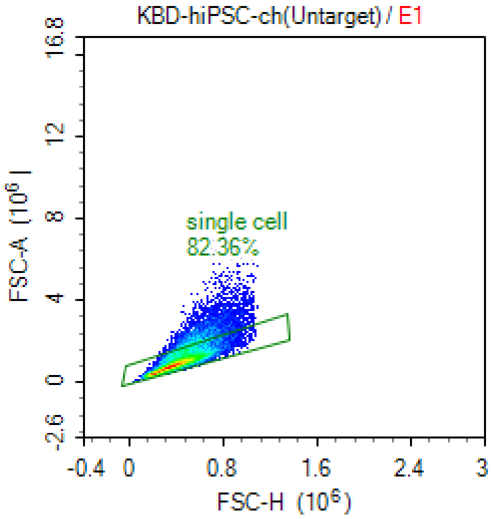

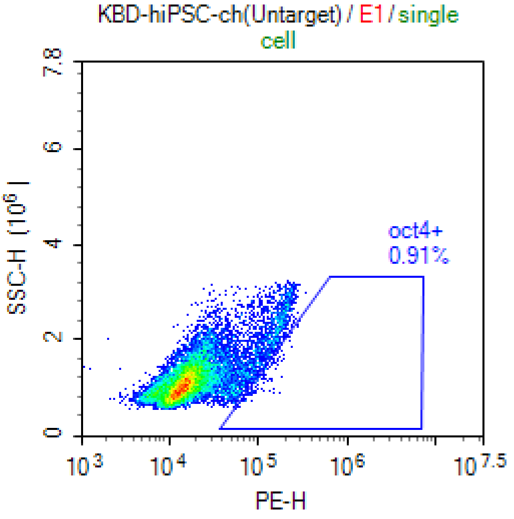

2. Isotype control

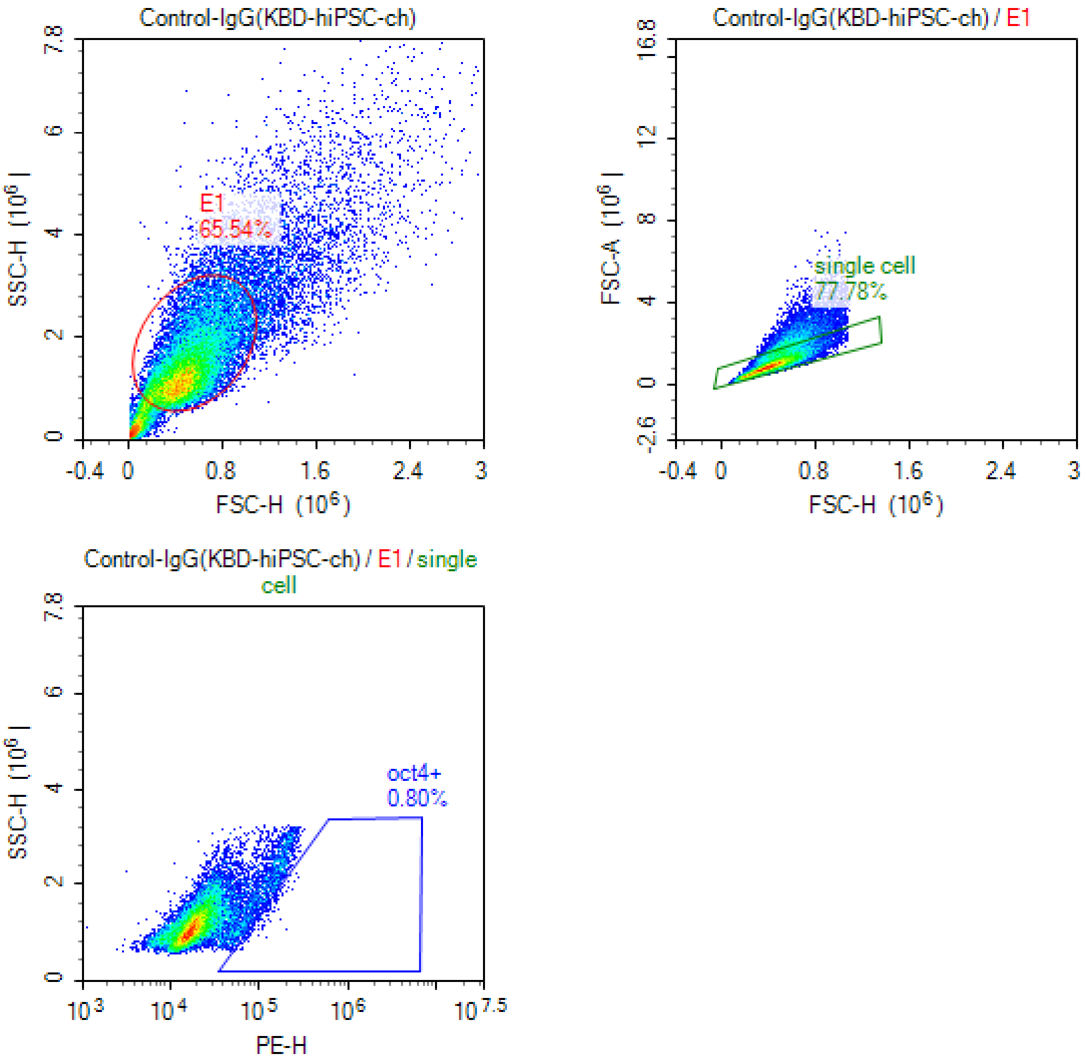
Control-IgG(KBD-hiPSC-ch)
E1
65.54%
SSC-H (10^6)
FSC-H (10^6)
Control-IgG(KBD-hiPSC-ch) / E1
single cell
77.78%
FSC-A (10^6)
FSC-H (10^6)
Control-IgG(KBD-hiPSC-ch) / E1 / single cell
oct4+
0.80%
SSC-H (10^6)
PE-H

3. OCT4+

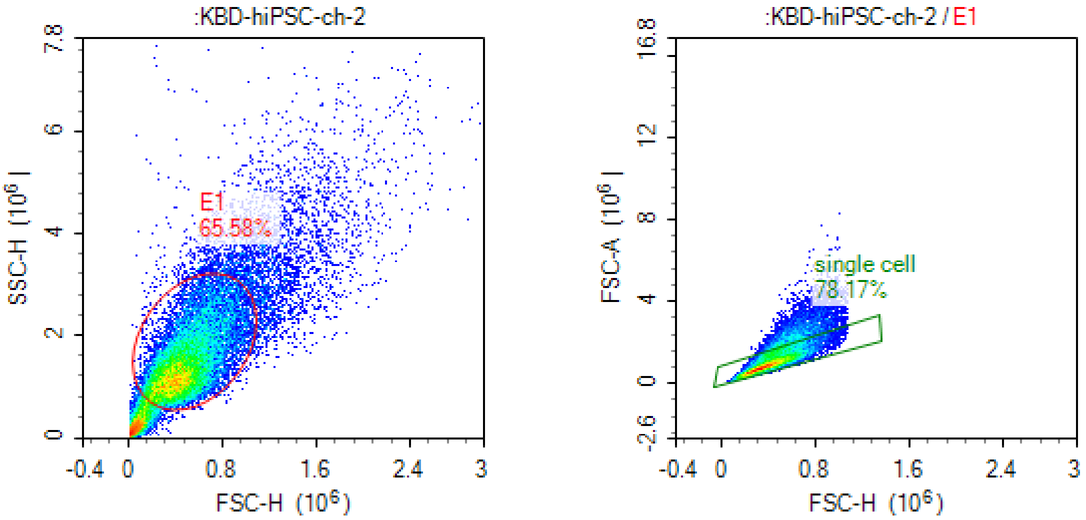
:KBD-hiPSC-ch-2
E1
65.58%
SSC-H (10^6)
FSC-H (10^6)
:KBD-hiPSC-ch-2 / E1
single cell
78.17%
FSC-A (10^6)
FSC-H (10^6)

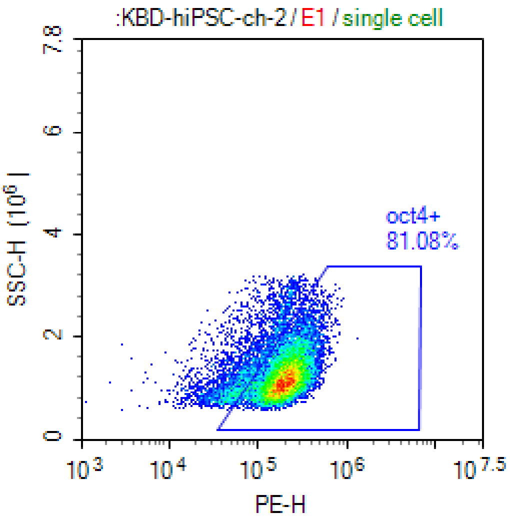
:KBD-hiPSC-ch-2 / E1 / single cell
oct4+
81.08%
SSC-H (10^6)
PE-H
0
2
4
6
7.8
10^3
10^4
10^5
10^6
10^7.5
