# Supplementary material for: Involvement of Yes-Associated Protein 1 Activation in the Matrix Degradation of Human-Induced-Pluripotent-Stem-Cell-Derived Chondrocytes Induced by T-2 Toxin and Deoxynivalenol Alone and in Combination
Source: Int J Mol Sci. 2024 Jan 10;25(2):878. doi: 10.3390/ijms25020878 (PMC10815455; doi:10.3390/ijms25020878)

Supplementary files: Original figures for Western Blotting.

Figure 2F:

Caption: from left to right:

Control, T-2, DON, T-2+DON   Control, T-2, DON, T-2+DON

***β*- actin (N-hiPS-Ch).**

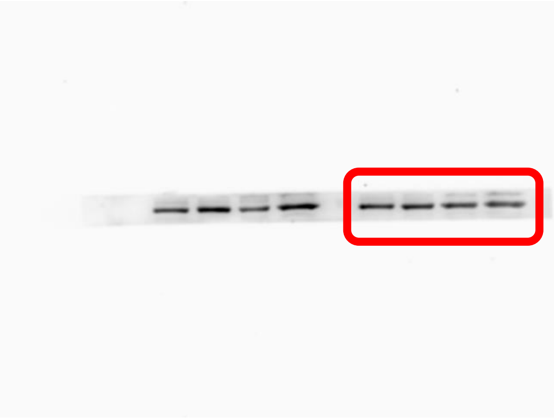

***β*- actin (KBD-hiPS-Ch).**

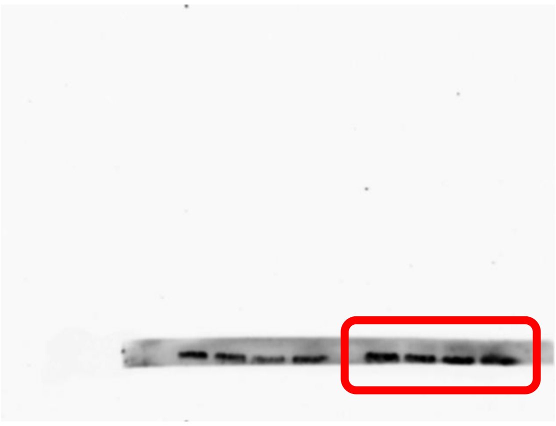

SOX9(N-hiPS-Ch)

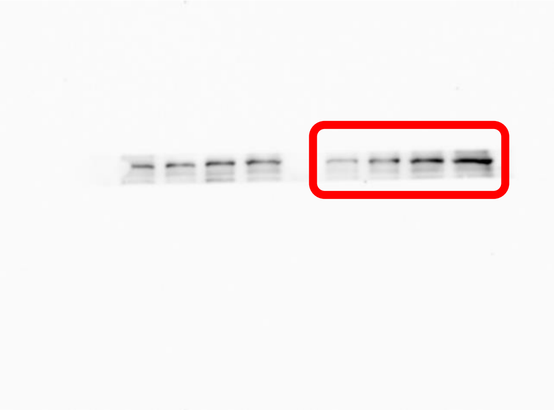

SOX9(KBD-hiPS-Ch).

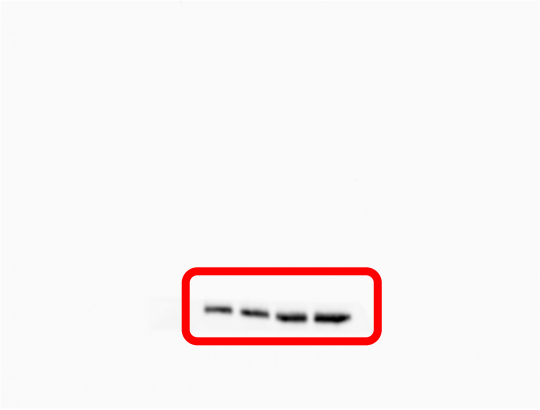

COL2A1(N-hiPS-Ch).

COL2A1(KBD-hiPS-Ch)

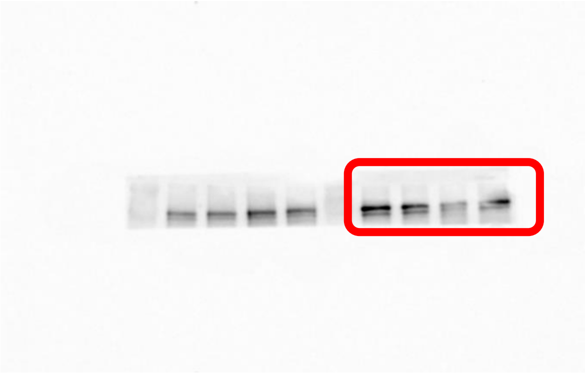

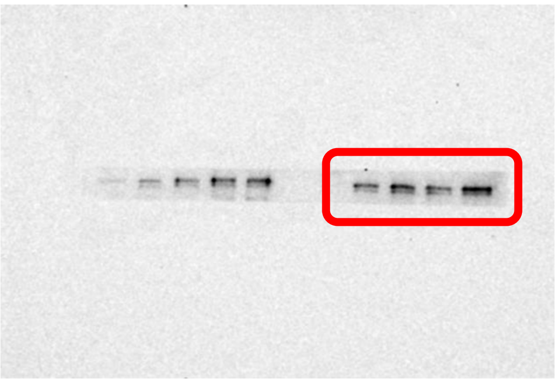

MMP13 (N-hiPS-Ch) MMP13(KBD-hiPS-Ch)

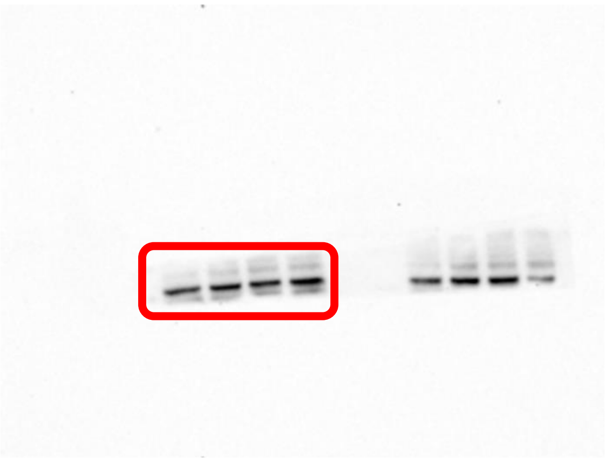

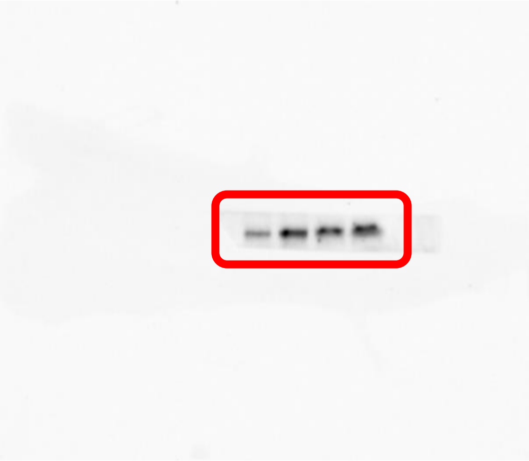

Figure 3F：

Caption: from left to right:

Control, T-2, DON, T-2+DON Control, T-2, DON, T-2+DON

β- actin(N-hiPS-Ch) ***β***- actin (KBD-hiPS-Ch)

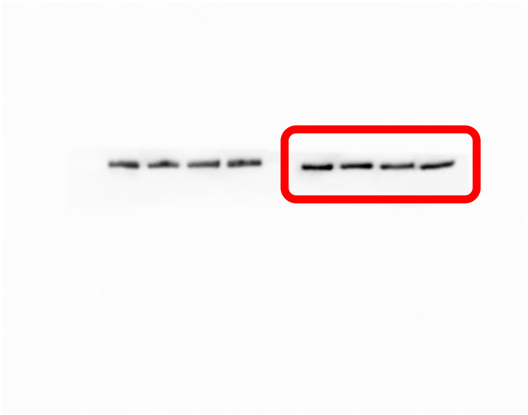

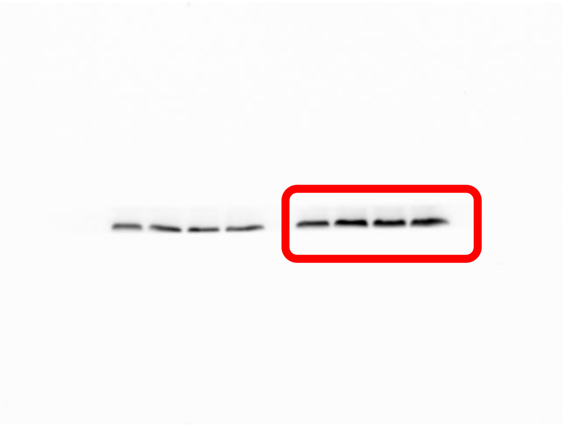

YAP (N-hiPS-Ch)

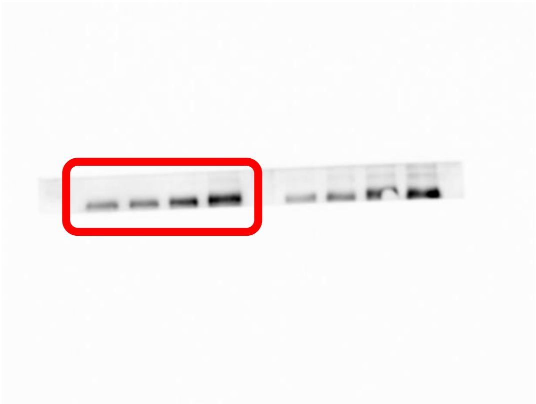

YAP (KBD-hiPS-Ch)

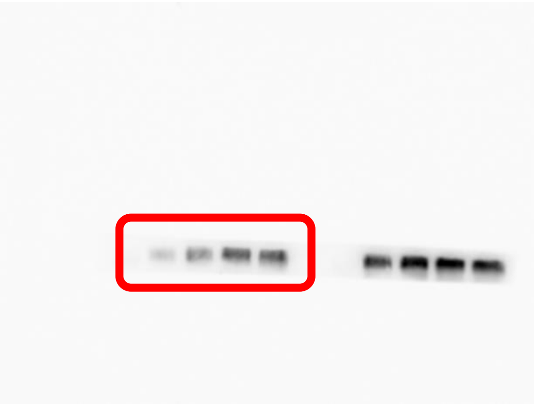

P-YAP(N-hiPS-Ch)

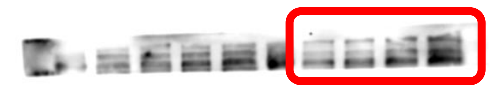

P-YAP(KBD-hiPS-Ch)

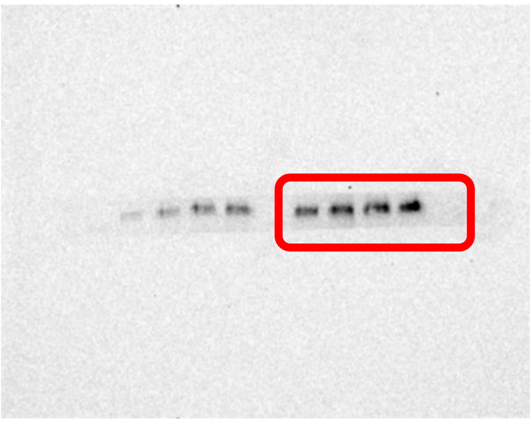

Figure 4F

Caption: from left to right:

Verteporfin(μmol/L) 0, 2, 5, 10 0, 2, 5, 10.

β- actin (N-hiPS-Ch)

β- actin (KBD-hiPS-Ch)

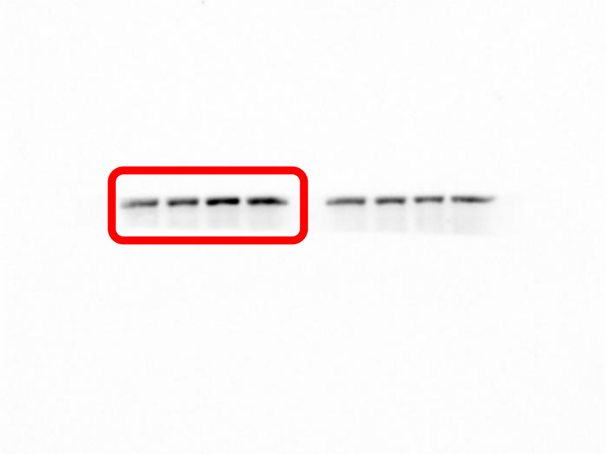

YAP(N-hiPS-Ch)

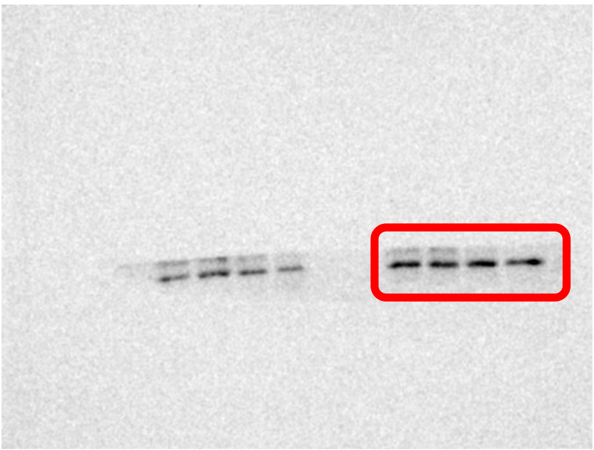

YAP(KBD-hiPS-Ch)

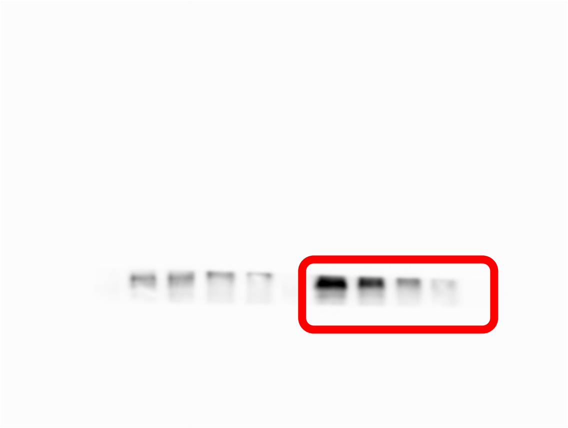

P-YAP (N-hiPS-Ch)

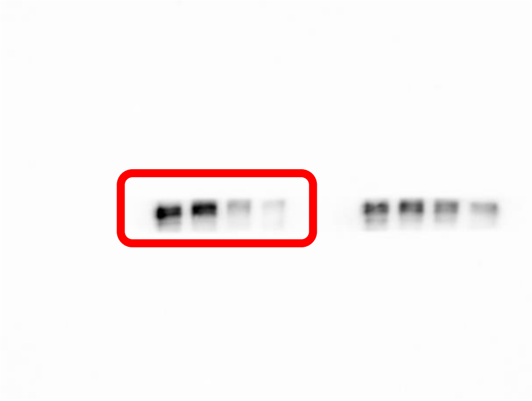

P-YAP (KBD-hiPS-Ch)

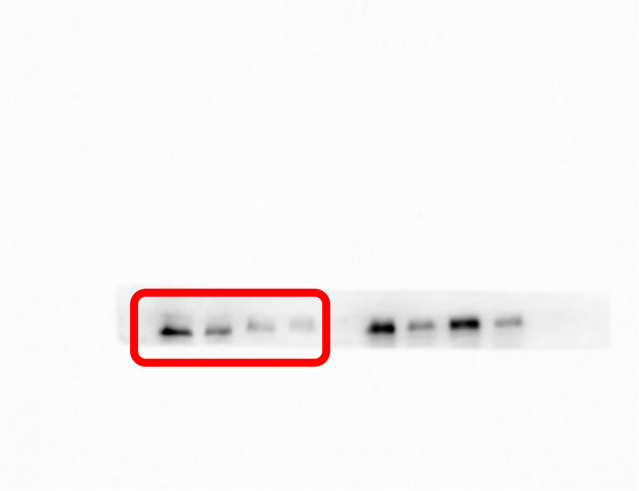

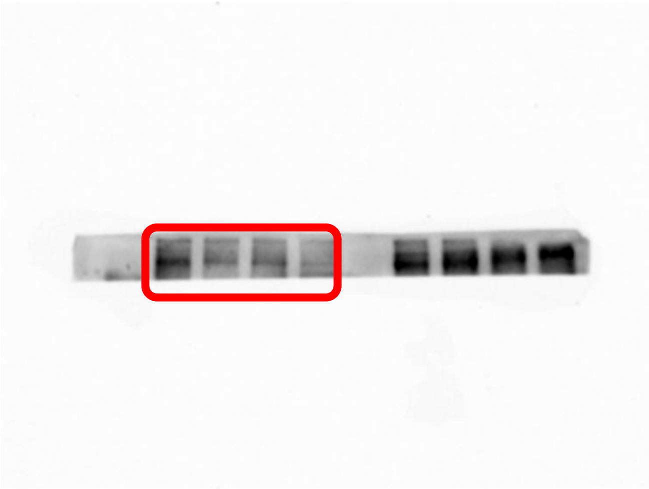

Figure 4G

Caption: from left to right:

Verteporfin(µmol/L) 0, 2, 5, 10   0, 2, 5, 10.

β- actin (N-hiPS-Ch)

β- actin (KBD-hiPS-Ch)

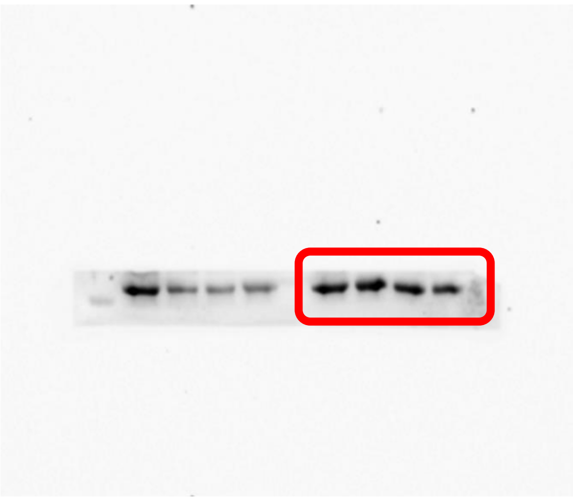

SOX9(N-hiPS-Ch)

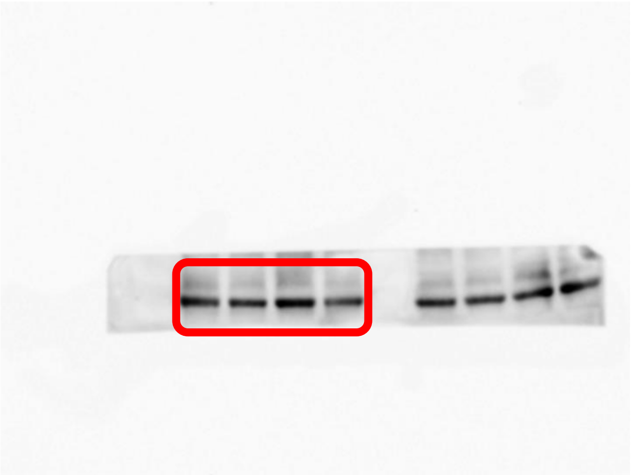

SOX9(KBD-hiPS-Ch)

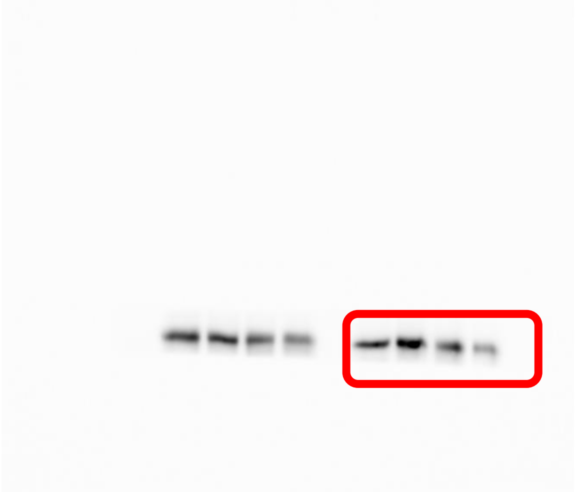

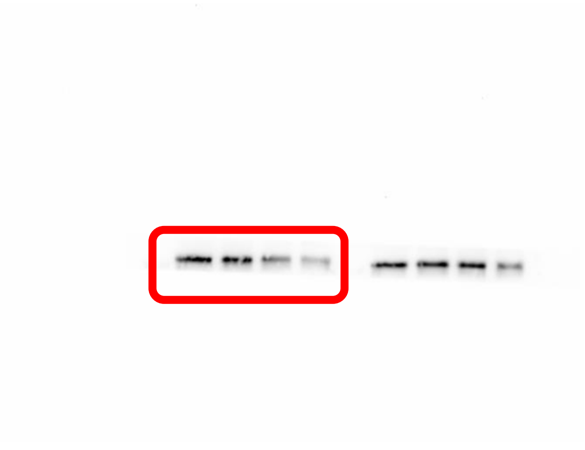

COL2A1(N-hiPS-Ch)

COL2A1(KBD-hiPS-Ch)

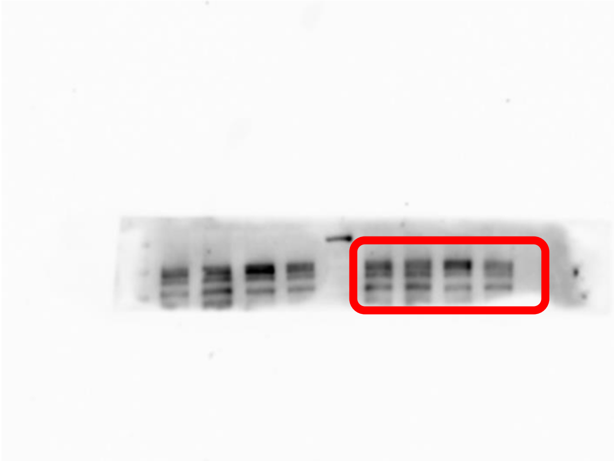

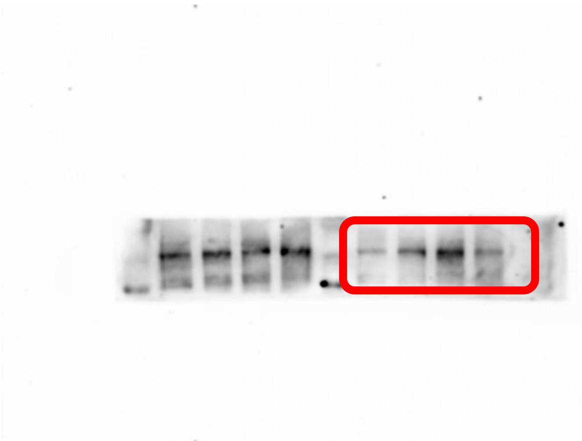

MMP13(N-hiPS-Ch)

MMP13(KBD-hiPS-Ch)

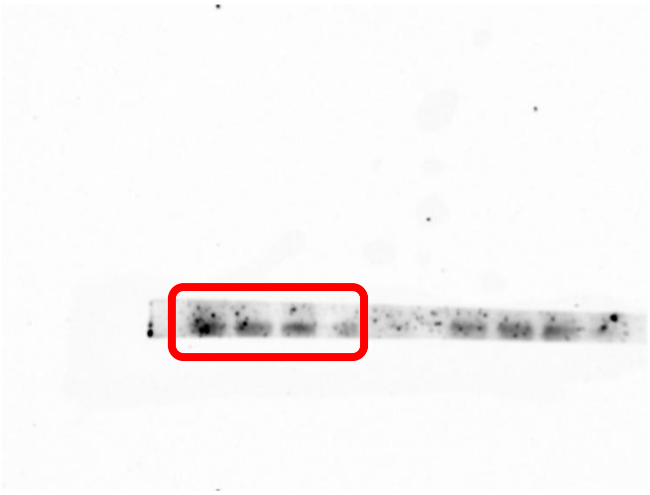

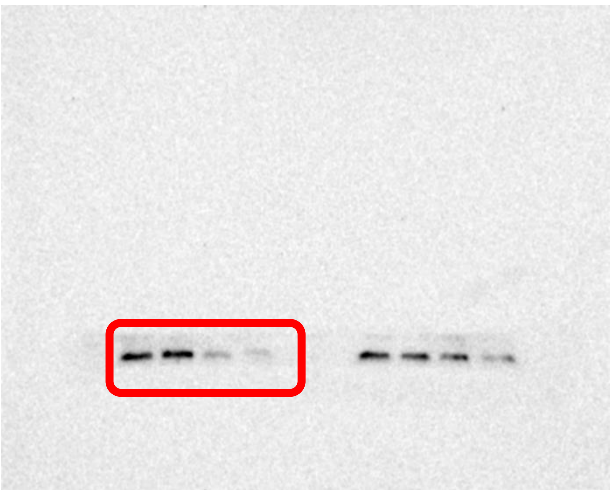

Supplement: Supplementary file 1 [file ijms-25-00878-s001.zip › Supplementary file S2.pdf]
